# Efficacy and safety of pegylated liposomal doxorubicin and epirubicin as neoadjuvant chemotherapy for breast cancer

**DOI:** 10.3389/fcell.2024.1448037

**Published:** 2024-12-23

**Authors:** Yuanyuan Shen, Qingling Hua, Menghao Dong, Wei Jin, Xueyang Hu, Kangsheng Gu

**Affiliations:** ^1^ Department of Medical Oncology, The First Affiliated Hospital of Anhui Medical University, Hefei, China; ^2^ Department of Medical Oncology, The First Affiliated Hospital of USTC, Division of Life Sciences and Medicine, University of Science and Technology of China, Hefei, China

**Keywords:** breast cancer, neoadjuvant chemotherapy, pegylated liposomal doxorubicin, epirubicin, efficacy

## Abstract

**Aim:**

This study aims to compare the efficiencies and toxicities of pegylated liposomal doxorubicin (PLD) based and epirubicin based chemotherapeutic regimens as neoadjuvant chemotherapy (NAC) for early breast cancer.

**Patients and methods:**

We retrospectively analyzed 391 patients with stage II-III breast cancer who received NAC in multiple centers. The efficiencies and toxicities of PLD and epirubicin based NAC regimens were compared by using both propensity-score matched (PSM) and unmatched data. The status of relevant gene loci was detected through next-generation sequencing (NGS) technology and the correlation between mutations of genes and serious adverse events (AEs) was preliminarily analyzed.

**Results:**

A total of 391 patients were included in this study. Among them, 235 patients received PLD based NAC (PLD group), and the other 156 patients were administrated with EPI based NAC (EPI group). The pathological complete response (pCR) rate of patients in PLD group was significantly higher than EPI group (before PSM:32.3% vs. 23.1%; after PSM:42.5% vs. 24.7%). Most severe AEs of patients in EPI group were more than PLD group (before PSM: 1.3%–37.8% vs. 0%–10.6%; after PSM: 1.4%–37.0% vs. 0%–9.6%). Mutation rates of 7 gene (MTHFR, DPYD, NQO1, ERCC1, UGT1A1, TYMS and TP53) of patients with severe AEs were significantly higher compared with patients with slight AEs (grade 1/2) or without AEs.

**Conclusion:**

PLD based chemotherapeutic regimen is a viable option for NAC of breast cancer. Epirubicin should be avoided for patients with mutations of some specific genes considering the potential severe AEs.

## Introduction

Breast cancer is one of the most common types of malignancies in the world (Siegel et al., 2022). During the past decades, advancements of therapies (i.e., surgery, chemotherapy, radiotherapy, and targeted therapy, etc.) significantly improve the prognosis of breast cancer (Swain et al., 2023). Among the therapies, neoadjuvant chemotherapy (NAC) plays a vital role especially in patients with locally advanced cancer. Until now, anthracycline based NAC regimens are effective and commonly used for breast cancer patients. Conventional anthracycline drugs include doxorubicin and epirubicin, unfortunately, both of them have many adverse events (AEs), such as nausea, vomiting, and hematologic toxicities. Moreover, cumulative dose-dependent irreversible cardiac toxicities (such as heart failure and arrhythmia) were fatal, and combinational usage of anti-HER2 treatment may enhance the risk of heart failure (Ryberg et al., 2008).

To ameliorate the outcomes of breast cancer patients, novel drugs with less toxicities need to be used. Recent years, pegylated liposomal doxorubicin (PLD), a novel PEGylated, liposomal formulation of doxorubicin, has been recommended as the first-line drug for advanced breast cancers (Gil-Gil et al., 2015). It is believed that PLD has comparable therapeutic effect to conventional anthracyclines but less severe AEs. Many studies indeed found that PLD and EPI based NAC had comparable clinical outcomes (e.g., pathological complete response, pCR) (Yang et al., 2018; Dong et al., 2018; Tsai et al., 2023). Therefore, it is necessary to further identify preferred patients who can significantly benefit from PLD. In addition, there are still many patients who choose epirubicin for various reasons, and it is also necessary to predict who may suffer serious AEs. The purpose of this study is to explore the characteristics of patients who can benefit from PLD treatment and who avoid severe AEs derived from epirubicin treatment.

## Methods

### Inclusion criteria

The study included patients with stage II-III primary breast cancer who received neoadjuvant chemotherapy. These patients received NAC treatments from January 2016 to December 2023 at the First Affiliated Hospital of Anhui Medical University and Anhui Provincial Hospital in Hefei, China. Inclusion criteria: (1) 18≤and≤75 years old. (2) Pathologically diagnosed breast cancer. (3) Stage II–III. (4) Received PLD-based NAC or epirubicin-based NAC. (5) Eastern Cooperative Oncology Group (ECOG) performance status: 0–2. (6) Adequate bone marrow functional reserve (total white blood cells ≥4.0 × 10^9^/L, platelets≥100 × 10^9^/L, neutrophils≥2.0 × 10^9^/L, hemoglobin≥90 g/L). (7) Left ventricular ejection fraction (LVEF) ≥50%. The exclusion criteria for the study included (1) <18 or >75 years old. (2) Metastatic cancer. (3) History of other malignant diseases. (4) Patients who are intolerant to neoadjuvant chemotherapy. Of the 391 included patients, 235 received PLD based NAC regimen (referred to as PLD group thereafter), and the other 156 patients were administrated with epirubicin based NAC regimen (referred to as EPI group thereafter). Clinical characteristics of patients were retrospectively extracted from medical records. Informed consent was waived by the ethics committee because this study is retrospective, and this study was approved by the ethics committees of Anhui Provincial Hospital (ethics number 2019KY086).

### Treatments and clinical assessments

The chemotherapy regimens and drug dose were determined according to the National Comprehensive Cancer Network (NCCN) guidelines (www.nccn.org). After NAC, patients underwent surgery. Radiation was indicated for patients if necessary according to the guidelines. Every patient was administrated with ultrasound and breast magnetic resonance imaging (MRI) for clinical evaluation. Core needle biopsy was performed for every patient before NAC. Pathologic evaluation included morphology and molecular features such as estrogen receptor (ER), progesterone receptor (PR), human epidermal growth factor receptor 2 (HER-2), Ki-67, etc. (representative images were showed as Supplementary Figure S1). Specifically, positive ER/PR was defined as ≥1% expression by immunohistochemical (IHC) staining. Patients with ER+ and/or PR + was considered as HR (hormone receptor) positive. HER-2 positive was defined as IHC 3 + or FISH test amplified. The standard threshold value of Ki-67 was 20%. High Ki-67 expression was defined as specimen with IHC score ≥20%, otherwise low Ki-67 expression.

Tumor size and regional lymph nodes were assessed with breast MRI every two NAC cycles. Clinical tumor response evaluation was performed by using the Response Evaluation Criteria in Solid Tumors Criteria version 1.1 (RECIST 1.1) (Eisenhauer et al., 2009). Pathological complete response was defined as no identification of residual cancer or *in situ* cancer in the surgical specimen of breast and regional lymph node. During the chemotherapies, laboratory tests including complete blood count and serum chemistry were performed for every patient weekly or more if necessary. Electrocardiogram was carried out every 3 weeks. Echocardiography was performed before the first NAC cycle, and re-measured every 3 chemotherapy cycles. After the completion of all NAC cycles, echocardiography was performed for every patient every 3 months at least for half a year. All AEs were assessed according to Common Terminology Criteria for Adverse Events (CTCAE) version 5.0 (Freites-Martinez et al., 2021). Adverse events were recorded at baseline and after each chemotherapy cycle.

### Next-generation sequencing

Patients’ baseline blood samples were used for next-generation sequencing. Genomic DNA was extracted from patients’ baseline blood samples. Genomic profiling was performed by using a panel of 425 cancer and drug metabolism related genes. A minimum of 50 ng of DNA with high‐quality was used to build NGS library. DNA shearing, end repair, phosphorylation, and adapter ligation were performed successively. Fragments (200–400 bp) were purified and hybridizated with capture probes baits, selected with magnetic beads, and amplificated. Following NGS library construction, indexed samples were sequenced by using paired‐end reads and an average sequencing depth of 1,000×. Whole exons and related introns of these genes were sequenced. The sequencing results were analyzed by bioinformatics to obtain severe AEs related mutant genes. BWA Picard mapped the reads to the reference human genome (hg19). The Genome Analysis Tool Kit and VarScan were used to identify gene mutations. The VarScan fpfilter pipeline was used to filter out loci with a depth of less than 100. Variant annotation was performed by using ANNOVAR and SnpEff.

### Statistical analysis

Statistical analyses were performed by using SPSS software (version 23.0). Propensity score matching (PSM) was performed to reduce potential selection bias between the PLD and EPI group, by considering patient characteristics including age, body mass index (BMI), menopausal status, tumor size, metastatic lymph nodes, clinical stage, histological grade, molecular types and Ki-67 expression. A 1:1 match was conducted by using the nearest neighbor within-caliper matching strategy with caliper of width equal to 0.2 between PLD and EPI group. Diferences between groups were compared using Pearson Chi-squared or Fisher’s exact test for categorical variables and *t*-test or Wilcoxon signed-rank test for continuous variables. A two-tailed P value of <0.05 was considered statistically significant.

## Results

### Clinical characteristics of included patients

**FIGURE 1:**
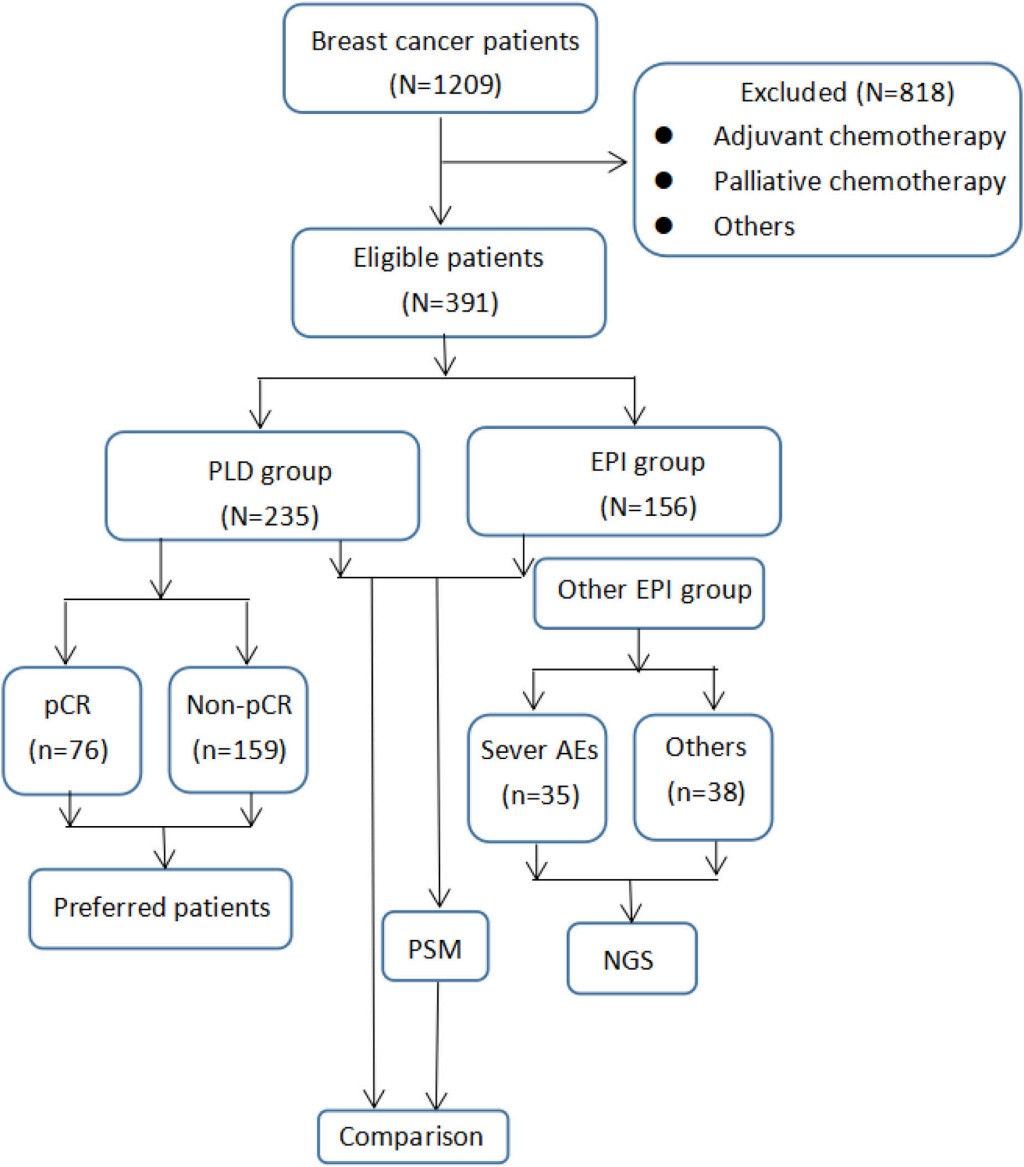
Flow chart of the present study.

As shown in the flow chart figure (Figure 1), 1,209 breast cancer patients were initially screened. However, 240 patients were not included in this study due to not receiving neoadjuvant chemotherapy. Other 400 patients were excluded because the patients received palliative chemotherapy. Fifty-eight patients were not included in the absence of detailed pathology reports. After that, 120 patients did not meet our inclusion criteria because they were over 75 years old. Finally, 391 patients were included in this study. Among them, 235 patients received PLD based NAC, and the other 156 patients were administrated with EPI based NAC. Detailed clinical features of included patients were listed in Table 1.

**TABLE 1:** Clinical characteristics of included patients.

Characteristics	Before PSM			After PSM		
	PLD (n = 235)	EPI (n = 156)	P-value	PLD (n = 146)	EPI (n = 146)	P-value
Age (M ± SD)	50.328 ± 9.213	48.628 ± 10.397	0.091	48.527 ± 9.357	48.795 ± 10.513	0.819
BMI	24.016 ± 3.000	24.295 ± 3.665	0.410	23.978 ± 2.807	24.312 ± 3.709	0.386
Menopausal status			0.014			0.810
Premenopausal	115	96		88	90	
Postmenopausal	120	60		58	56	
Clinical tumor staging			0.824			0.733
1	29	16		16	15	
2	168	110		108	102	
3	31	25		19	24	
4	7	5		3	5	
Clinical N staging			0.010			0.995
0	21	22		21	22	
1	139	100		92	90	
2	18	16		16	16	
3	57	18		17	18	
Clinical TNM staging			0.061			0.800
II	144	110		102	100	
III	91	46		44	46	
Histological grade			0.010			
1	4	9		4	9	0.211
2	158	115		101	105	
3	73	32		41	32	
Molecular types			0.839			0.539
HR + HER-2+	52	32		38	30	
HR + HER-2-	124	83		71	76	
HR-HER-2+	21	11		14	11	
HR-HER-2-	38	29		23	29	
Ki-67			0.190			0.544
<20%	23	22		12	15	
≥20%	212	134		134	131	

PSM, propensity score matching; BMI, body mass index; HR, hormone receptor, including ER an PR; HER-2, human epidermal growth factor receptor 2.

### Efficacies of PLD and EPI based NAC

**FIGURE 2:**
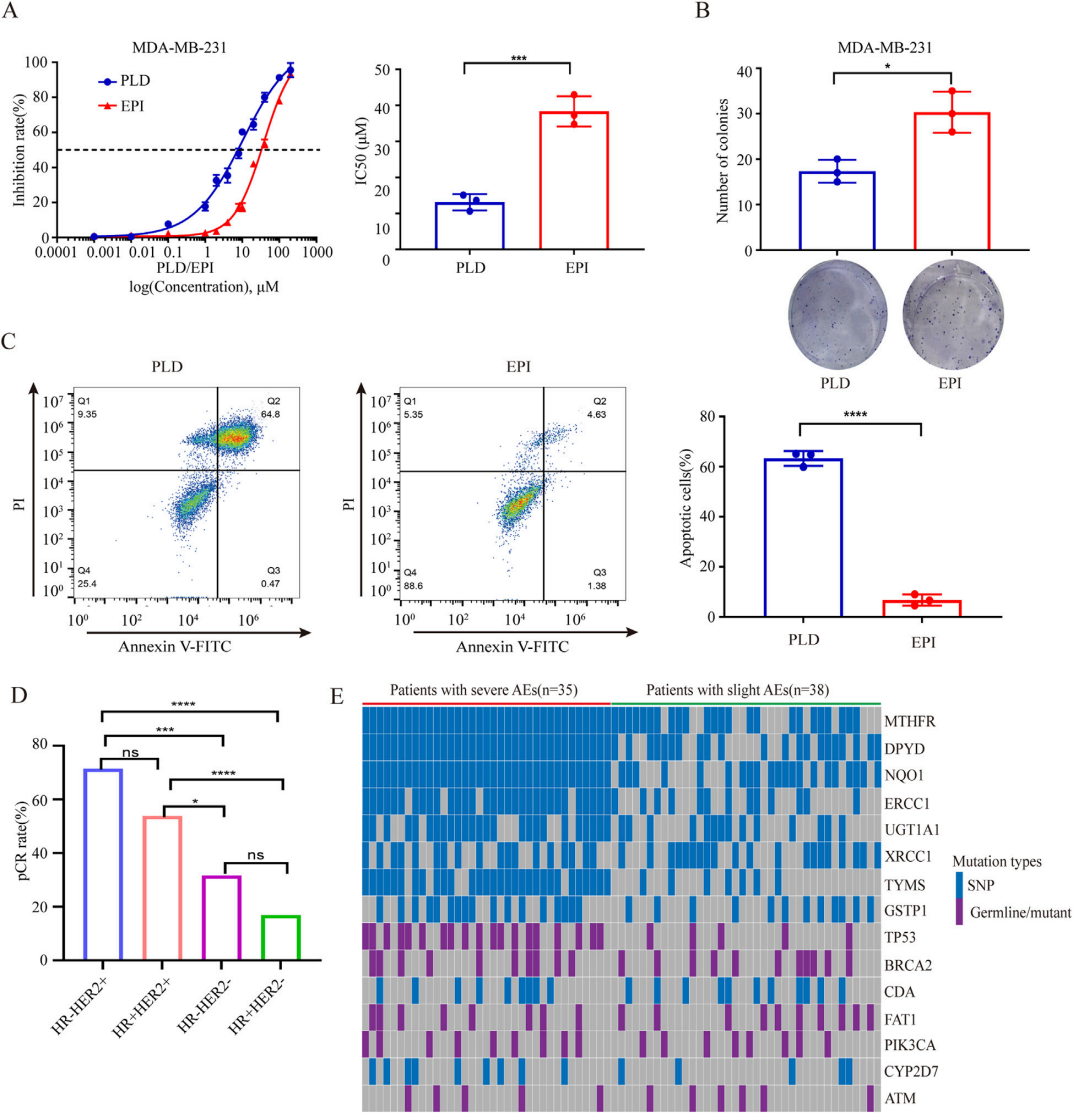
**(A)** Comparisons of IC50 of PLD and EPI in breast cancer cells. **(B)** Comparisons of clonal abilities of breast cancer cells undergone administrations of PLD and EPI. **(C)** Comparisons of apoptosis rates of breast cancer cells undergone administrations of PLD and EPI. **(D)** Comparisons of pCR rates of PLD administrated patients with different molecular features. **(E)** Heatmap of mutated genes of patients with severe or slight AEs. *P < 0.05, ***P < 0.001, ****P < 0.0001. ns, not significant.

We compared the pCR rates between PLD and EPI group and found that the pCR rate of patients in PLD group was significantly higher than EPI group regardless of PSM (before PSM:32.3% vs. 23.1%, *p* = 0.047; after PSM:42.5% vs. 24.7%, *p* = 0.001). Before PSM, no statistical differences of clinical response were identified between the groups. However, after PSM, ORR of PLD group was higher than EPI group (82.9% vs. 69.9%, *p* = 0.018). Moreover, higher Miller-Payne grades (grade V) were achieved in PLD group (before PSM:34.5% vs. 24.4%, *p* = 0.033; after PSM:43.8% vs. 26.0%, *p* = 0.001) (Table 2). *In vitro* experiments, we compared the cytotoxicity of PLD and EPI in breast cancer cells and found that the IC50 of PLD was significant lower than EPI. Colony and apoptosis experiments were also performed and the results verified the higher cytotoxicity of PLD compared with EPI (Figures 2A–C). To explore the preferred patients for PLD, we compared the clinical features of patients with pCR and non-pCR in PLD group. Interestingly, patients with HR-HER2 + subtype have the highest pCR rate (15/21, 71.4%) compared with other molecular subtypes. Patients with HR + HER2 + pathological feature have the second highest pCR rate (28/52, 53.8%) (Table 3) (Figure 2D). It should be noted that many patients with positive HER-2 feature received NAC regimens including trastuzumab. We believed that the higher pCR rate of HER-2 positive patients mainly derive from the administration of anti-HER-2 therapy.

**TABLE 2:** Comparisons of outcomes of patients.

	Before PSM			After PSM		
	PLD (n=235)	EPI (n=156)	P-value	PLD (n=146)	EPI (n=146)	P-value
Pathological response			0.047			0.001
pCR^a^	76(32.3 %)	36(23.1%)		62(42.5%)	36(24.7%)	
Non-pCR	159(67.7%)	120(76.9%)		84(57.5%)	110(75.3%)	
Radiologicalresponse			0.130			0.018
ORR(CR+PR)	188(80.0%)	111 (71.2%)		121(82.9%)	102(69.9%)	
SD	45(19.1%)	43(27.6%)		23(15.8%)	43(29.5%)	
PD	2(0.9%)	2(1.2%)		2(1.3%)	1(0.6%)	
Miller-Payne grade			0.033			0.001
I-IV	154(65.5%)	118(75.6%)		82(56.2%)	108(74.0%)	
V	81(34.5%)	38(24.4%)		64(43.8%)	38(26.0%)	

PSM, propensity score matching; CR, complete response; PR, partial response; ORR, overall response rate; SD, stable disease; PD, progressed disease.

^a^ There are two patients were diagnosed as in situ cancer in PLD group with pCR before PSM.

No patients have in situ cancer in EPI group with pCR before PSM or both group after PSM.

**TABLE 3:** Prognostic factors of outcomes of patients in PLD group.

Characteristics	pCR (n = 76)	Non-pCR (n = 159)	P-value
Age	49.605 ± 8.783	50.673 ± 9.420	0.407
BMI	24.535 ± 2.800	23.768 ± 3.069	0.067
Menopausal status			0.740
Premenopausal	36 (47.4%)	79 (49.7%)	
Postmenopausal	40 (52.6%)	80 (50.3%)	
Clinical tumor staging			0.060
1	15 (19.7%)	14 (8.8%)	
2	53 (69.8%)	115 (72.3%)	
3	7 (9.2%)	24 (15.1%)	
4	1 (1.3%)	6 (3.8%)	
Clinical N staging			0.102
0	7 (9.2%)	14 (8.8%)	
1	48 (63.2%)	91 (57.2%)	
2	9 (11.8%)	9 (5.7%)	
3	12 (15.8%)	45 (23.3%)	
Clinical TNM staging			0.326
II	50 (65.8%)	94 (59.1%)	
III	26 (34.2%)	65 (40.9%)	
Histological grade			0.880
1	1 (1.3%)	3 (1.9%)	
2	50 (65.8%)	108 (67.9%)	
3	25 (32.9%)	48 (30.2%)	
HR			0.011
Negative	27 (35.5%)	32 (20.1%)	
Positive	49 (64.5%)	127 (79.9%)	
HER2			<0.0001
Negative	33 (43.4%)	129 (81.1%)	
Positive	43 (56.6%)	30 (18.9%)	
Molecular types			<0.0001
HR + HER2+	28 (36.8%)	24 (15.2%)	
HR + HER2-	21 (27.6%)	103 (64.8%)	
HR-HER2+	15 (19.7%)	6 (3.8%)	
HR-HER2-	12 (15.8%)	26 (16.4%)	
Ki67			0.011
<20%	2 (2.6%)	21 (13.2%)	
≥20%	74 (97.4%)	138 (86.8%)	

BMI, body mass index; HR, hormone receptor.

### Toxicities of PLD and EPI based NAC

Most common AEs of patients included non-cardiac toxicities (e.g., leukopenia, anemia, thrombocytopenia, nausea/vomiting, mucositis and alopecia) and cardiac toxicities (i.e., abnormal ECG and declined left ventricular ejection fractions). As shown in Table 4, most severe AEs (grade 3/4) of patients in EPI group were more than PLD group (before PSM: 1.3%–37.8% vs. 0%–10.6%; after PSM: 1.4%–37.0% vs. 0%–9.6%). Even previous studies indicated that PLD based NAC was better than EPI based NAC, many patients choose EPI in current clinic practice. Considering the evidently severe AEs derived from EPI, a novel tool need to be developed to identify the susceptible patients. Published studies demonstrated that chemotherapy related AEs have strong relationships with mutant oncogene and drug metabolic genes. To explore the genes correlated with toxicities of EPI based NAC, we recruited patients with plan to receive EPI based NAC and collected their blood samples for NGS (Supplementary Table S1). Total of 35 patients undergone severe AEs (grade 3/4) and 38 patients had slight AEs (grade 1/2) according to CTCAE (version 5.0). The analyses of NGS results showed that many mutations involved in metabolism and DNA repair related genes, such as UGT1A1, CYP2D7, BRCA2, etc (Table 5; Figure 2E). Moreover, mutation rates of 7 gene (MTHFR, DPYD, NQO1, ERCC1, UGT1A1, TYMS and TP53) were significantly higher of patients with severe AEs (grade 3/4) compared with patients with slight AEs (grade 1/2) (Table 6).

**TABLE 4:** Severe toxicities (Grade 3/4) of included patients.

	Before PSM			After PSM		
	PLD (n = 235)	EPI (n = 156)	P Value	PLD (n = 146)	EPI (n = 146)	P Value
Non-cardiac toxicities						
Leukopenia	9 (3.8%)	23 (14.7%)	0.000	7 (4.8%)	22 (15.1%)	0.003
Anemia	10 (4.3%)	19 (12.2%)	0.003	6 (4.1%)	18 (12.3%)	0.011
Thrombocytopenia	3 (1.3%)	11 (7.1%)	0.003	3 (2.1%)	11 (7.5%)	0.028
Nausea/Vomiting	6 (2.5%)	19 (12.2%)	0.000	4 (2.7%)	19 (13.0%)	0.001
Mucositis	4 (1.7%)	3 (1.9%)	0.851	3 (2.1%)	2 (1.4%)	0.652
Alopecia	25 (10.6%)	59 (37.8%)	0.000	14 (9.6%)	54 (37.0%)	0.000
Cardiac toxicities						
Abnormal ECG	3 (1.3%)	9 (5.7%)	0.012	2 (1.4%)	8 (5.7%)	0.054
LVEF decline >10%	0 (0%)	2 (1.3%)	0.082	0 (0%)	3 (2.1%)	0.082

PSM, propensity score matching; ECG, electrocardiogram; LVEF, left ventricular ejection fractions.

**TABLE 5:** Most common mutated genes.

Genes	Number of patients	Type
MTHFR	59	SNP
DPYD	56	SNP
NQO1	56	SNP
ERCC1	44	SNP
UGT1A1	39	SNP
XRCC1	38	SNP
TYMS	37	SNP
GSTP1	29	SNP
TP53	24	Germline/Mutant
BRCA2	21	Germline/Mutant
CDA	20	SNP
FAT1	18	Germline/Mutant
PIK3CA	17	Germline/Mutant
CYP2D7	14	SNP
ATM	11	Germline

SNP, single nucleotide polymorphism.

**TABLE 6:** Comparison of gene mutations of patients with severe and slight AEs.

Genes	Severe AEs (n = 35)	Slight AEs (n = 38)	P-value
MTHFR	35 (100%)	24 (63.2%)	<0.0001
DPYD	35 (100%)	21 (55.3%)	<0.0001
NQO1	35 (100%)	21 (55.3%)	<0.0001
ERCC1	31 (88.6%)	13 (34.2%)	<0.0001
UGT1A1	25 (71.4%)	14 (36.8%)	0.003
XRCC1	18 (51.4%)	20 (52.6%)	0.598
TYMS	30 (85.7%)	7 (18.4%)	0.000
GSTP1	17 (48.6%)	12 (31.6%)	0.138
TP53	19 (54.3%)	5 (13.2%)	0.000
BRCA2	10 (28.6%)	11 (28.9%)	0.972
CDA	9 (25.7%)	11 (28.9%)	0.757
FAT1	8 (22.9%)	10 (26.3%)	0.732
PIK3CA	9 (25.7%)	8 (21.1%)	0.638
CYP2D7	9 (25.7%)	5 (13.1%)	0.173
ATM	5 (14.3%)	6 (15.8%)	0.858

AE, adverse events.

## Discussion

This study compared PLD based and EPI based NAC directly, and distinguished preferred patients for each regimen. The results showed that the pCR rate of patients received PLD based NAC was significantly higher than EPI based regimen. Moreover, we found that HER2 expressed patients with PLD administration had higher pCR rate than other patients. As for toxicities, most severe AEs of patients in EPI group were more than PLD group. Fortunately, mutation rates of 7 gene (MTHFR, DPYD, NQO1, ERCC1, UGT1A1, TYMS and TP53) had potential to predict the occurrence of severe AEs. Overall, this study identified preferred breast cancer patients for PLD or epirubicin based regimens used as NAC.

Epirubicin has been widely used in NAC regimen for invasive breast cancer. However, many clinical trials showed that the use of epirubicin is partially restricted due to severe AEs (Schneeweiss et al., 2022). Trastuzumab/pertuzumab (HP) combination has become the cornerstone of neoadjuvant chemotherapy for breast cancer patients with HER2 overexpression, the combination of HP and EPI chemotherapy regimen can increase cardiac toxicity. It is proved that PLD provides a substitute for epirubicin for patients with metastatic breast cancer. In recent years, the use of PLD for NAC of breast cancer is growing. A Phase II study reported in 2002 indicated that PLD was effective in NAC for breast cancer and its toxicity was manageable (Golshan et al., 2015). Likewise, Manguso et al. reported satisfactory efficacy and low rate of toxicities of PLD base NAC in operable and locally advanced breast cancer (Manguso et al., 2015). Several previous studies demonstrated that patients administrated with PLD have better pCR rate compared to conventional anthracyclines, which was consistent with our study (Tsai et al., 2023). Hung et al. reported that the PLD-based and epirubicin-based NAC have similar pCR, recurrence, and overall survival rate (Hung et al., 2020). Drug delivery system of liposomes could increase the size of drug molecule and improve the accumulation of hemotherapeutic agents in the tumor microenvironment (Torchilin, 2011), which may partly explains the diferences in treatment response between PLD and epirubicin-based NAC in our study.

Tsai et al.‘s study indicated that HER2-enriched patients have a higher pCR rate than other patients. In our study, HR-HER2+ patients have the highest pCR rate (71.4%) compared with other molecular subtypes. Patients with HR + HER2+ pathological feature have the second highest pCR rate (53.8%). We believed that the higher pCR rate of HER2-enriched patients derived from anti-Her2 target therapy. However, Li et al. reported that patients with triple negative breast cancer (TNBC) subtype had the highest pCR rate (43.8%) among the different molecular types (Li et al., 2019). The difference between Li et al.‘s study and ours may result from the different sample size (235 vs. 97).

In this study, pCR serves as the primary endpoint, however, it is controversial to evaluate the efficacy of NAC regimens by using pCR. Several published studies indicated that pCR predicted good prognosis. Contrarily, a review and another meta-analysis show that pCR can be used for assessment of efficacy of treatment but it has no correlation with survival (Berruti et al., 2014; Cortazar et al., 2014). In our study, we failed to provide survival information, but compared the efficacy of PLD and epirubicin based NAC. In the further study, we would like to compare these two regimens in terms of long-term survival.

Patients treated with PLD experienced more leukopenia (grade 1/2) than those treated with epirubicin (54.8% vs. 32.9%, *p* < 0.0001). However, the severe leukopenia (grade 3/4) occurred more often (15.1% vs. 4.8%, *p* = 0.003) in patients of EPI group than PLD group. Likewise, other many severe adverse events (e.g., anemia, thrombocytopenia, nausea/Vomiting, etc.) were recorded more in EPI group. For cardiac toxicities, no significant difference was found between PLD and EPI group. Whether the gene mutation is related to the adverse reaction of chemotherapy for breast cancer is still in the exploration stage. There has been some evidence that neutropenia after chemotherapy is more common in patients with BRCA1/2 mutations (32% vs. 10%), and patients with BRCA1/2 mutations tend to be more sensitive to ionizing radiation, because BRCA gene is involved in DNA repair and cell cycle control mechanisms (Huszno et al., 2013; Pierce et al., 2000). In clinical practice, several genes were used to predict the efficacy and toxicity of drugs. For example, the UDP Glucuronosyltransferase Family 1 Member A1 (UGT1A1) gene encodes enzyme performing a chemical reaction called glucuronidation, in which a compound called glucuronic acid is attached to substance and the gene status of UGT1A1 can be detected to predict the toxicities of irinotecan (Jiang et al., 2024). In our study, besides UGT1A1, we identified 7 genes (MTHFR, DPYD, NQO1, ERCC1, UGT1A1, TYMS and TP53) correlated with severe toxicities derived from EPI based NAC. MTHFR (methylenetetrahydrofolate reductase) converts vitamin B9 (folate) into methyl-folate, which is essential for a biological process named methylation (Blomgren et al., 2024)^.^ DPYD (dihydropyrimidine dehydrogenase) is involved in pyrimidines metabolism, and have tight relationships with fluoropyrimidine (5-fluorouracil, capecitabine, and other analogs) metabolism (De Metz et al., 2024). NQO1 [NAD (P) H quinone dehydrogenase 1] encodes a cytoplasmic 2-electron reductase and reduces quinones to hydroquinones. Mutations in this gene have been associated with tardive dyskinesia (TD), an increased risk of hematotoxicity after exposure to benzene, and susceptibility to various forms of cancer (Okumura et al., 2021). ERCC1 (Excision Repair Cross Complementation Group 1) encodes protein for nucleotide excision repair, and is required for the repair of DNA lesions such as those induced by UV light or formed by electrophilic compounds including cisplatin (Blee et al., 2024). TYMS (Thymidylate synthase) catalyzes the methylation of deoxyuridylate to deoxythymidylate, which involved in DNA replication and repair (Nguyen et al., 2024). The TP53 gene provides instructions for making a tumor suppressor, p53, and the mutation of TP53 may results in oncogenesis (Chauhan et al., 2024). Most of these genes involved in metabolism, we believed that mutation of these genes tend to affect the metabolism of epirubicin and lead to severe AEs. Patients with these genes mutation should avoid administration of epirubicin for NAC of breast cancer.

In this study, we reported both unmatched and propensity-score matched data to minimize confounding factors. A propensity score is the probability that a unit with certain features will be assigned to the experimental or control group. The scores can be used to reduce selection bias in observational studies. By using this statistical method, the validity and robustness of our inclusions could be strengthened. However, it is worth noting that there are several limitations in the current analysis. Firstly, although PSM was employed to reduce selection bias, the retrospective design still leaves room for unmeasured confounding factors that may produce unreliable results. Secondly, the retrospective nature of this present study and absence of survival information may restrict the generalization and validity of our findings. In other words, our findings may not apply to all breast cancer patients, which should be noted.

## Conclusion

PLD based regimen is a viable option for NAC of breast cancer, especially for patients with HER-2 overexpressing pathological feature. Epirubicin should be avoided for patients with mutations of some specific genes considering the potential severe AEs. Further prospective study with larger sample size and longer follow-up duration should be conducted to evaluate the advantages of PLD for NAC.

## Data Availability

The raw data supporting the conclusions of this article will be made available by the authors, without undue reservation.
