# Supplementary material for: Efficacy and safety of pegylated liposomal doxorubicin and epirubicin as neoadjuvant chemotherapy for breast cancer
Source: Front Cell Dev Biol. 2024 Dec 23;12:1448037. doi: 10.3389/fcell.2024.1448037 (PMC11701144; doi:10.3389/fcell.2024.1448037)
Supplement: Supplementary file 1 [file Table1.docx]

**Supplementary Table 1. Clinical features of patients receive EPI based NAC for NGS analyses**

| **Characteristics** |  | **EPI(n=73)** |
| --- | --- | --- |
| **Age**(M±SD) |  | 46.869±11.261 |
| **BMI** |  | 25.185±2.217 |
| **Menopausal status** |  |  |
| Premenopausal |  | 37 |
| Postmenopausal |  | 36 |
| **Clinical tumor staging** |  |  |
| 1 |  | 10 |
| 2 |  | 52 |
| 3 |  | 9 |
| 4 |  | 2 |
| **Clinical N staging** |  |  |
| 0 |  | 9 |
| 1 |  | 44 |
| 2 |  | 8 |
| 3 |  | 12 |
| **Clinical TNM staging** |  |  |
| II |  | 55 |
| III |  | 18 |
| **Histological grade** |  |  |
| 1 |  | 2 |
| 2 |  | 24 |
| 3 |  | 47 |
| **Molecular types** |  |  |
| HR+HER-2+ |  | 7 |
| HR+HER-2- |  | 48 |
| HR-HER-2+ |  | 5 |
| HR-HER-2- |  | 13 |
| **Ki-67** |  |  |
| <20% |  | 8 |
| ≥20% |  | 65 |

BMI:body mass index; HR: hormone receptor, including ER an PR; HER-2:human epidermal growth factor receptor 2
