# Supplementary figures and images for: Efficacy and safety of pegylated liposomal doxorubicin and epirubicin as neoadjuvant chemotherapy for breast cancer
Source: Front Cell Dev Biol. 2024 Dec 23;12:1448037. doi: 10.3389/fcell.2024.1448037 (PMC11701144; doi:10.3389/fcell.2024.1448037)

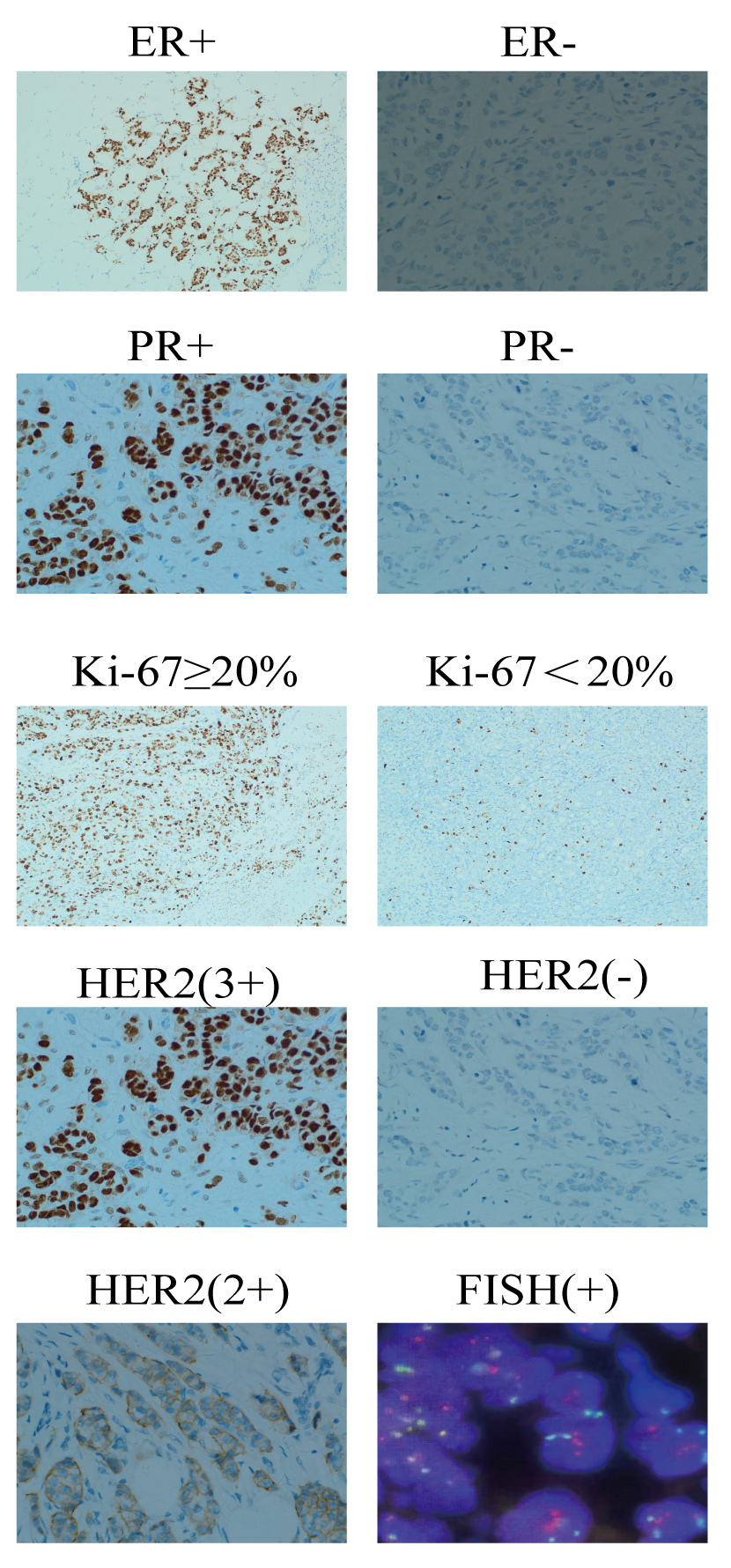

Supplement: Supplementary file 2 [file Image1.tif]
